# A Systematic Review and Meta-Analysis on the Significance of TIGIT in Solid Cancers: Dual TIGIT/PD-1 Blockade to Overcome Immune-Resistance in Solid Cancers

**DOI:** 10.3390/ijms221910389

**Published:** 2021-09-27

**Authors:** Negar Hosseinkhani, Mahdi Abdoli Shadbad, Mohammad Asghari Jafarabadi, Noora Karim Ahangar, Zahra Asadzadeh, Seyede Momeneh Mohammadi, Parisa Lotfinejad, Nazila Alizadeh, Oronzo Brunetti, Rossella Fasano, Nicola Silvestris, Behzad Baradaran

**Affiliations:** 1 Immunology Research Center, Tabriz University of Medical Sciences, Tabriz 5165665811, Iran; negar_hosseinkhani@yahoo.com (N.H.); nura_karimi@yahoo.com (N.K.A.); Zahraasadzadeh2834@gmail.com (Z.A.); p.lotfinezhad@gmail.com (P.L.); alizadeh_imm@yahoo.com (N.A.); 2 Research Center for Evidence-Based Medicine, Faculty of Medicine, Tabriz University of Medical Sciences, Tabriz 5166614766, Iran; abdoli.med99@gmail.com; 3 Department of Statistics and Epidemiology, School of Medicine, Zanjan University of Medical Sciences, Zanjan 4513956184, Iran; m.asghari862@gmail.com; 4 Center for the Development of Interdisciplinary Research in Islamic Sciences and Health Sciences, Tabriz University of Medical Sciences, Tabriz 4513956184, Iran; 5 Department of Anatomical Sciences, School of Medicine, Zanjan University of Medical Sciences, Zanjan 4513956184, Iran; mohamady3@gmail.com; 6 Medical Oncology Unit, IRCCS Istituto Tumori “Giovanni Paolo II” of Bari, 70124 Bari, Italy; dr.oronzo.brunetti@tiscali.it (O.B.); rossella.fasano.93@gmail.com (R.F.); 7 Department of Biomedical Sciences and Human Oncology (DIMO), University of Bari, 70124 Bari, Italy; 8 Department of Immunology, Tabriz University of Medical Sciences, Tabriz 5165665811, Iran; 9 Pharmaceutical Analysis Research Center, Tabriz University of Medical Sciences, Tabriz 5166614766, Iran

**Keywords:** TIGIT, PD-1, dual blockade, immune-resistance, immune checkpoint, solid cancers

## Abstract

Preclinical studies have indicated that T-cell immunoglobulin and ITIM domain (TIGIT) can substantially attenuate anti-tumoral immune responses. Although multiple clinical studies have evaluated the significance of TIGIT in patients with solid cancers, their results remain inconclusive. Thus, we conducted the current systematic review and meta-analysis based on the preferred reporting items for systematic reviews and meta-analyses (PRISMA) to determine its significance in patients with solid cancers. We systematically searched the Web of Science, Embase, PubMed, and Scopus databases to obtain peer-reviewed studies published before September 20, 2020. Our results have shown that increased TIGIT expression has been significantly associated with inferior overall survival (OS) (HR = 1.42, 95% CI: 1.11–1.82, and *p*-value = 0.01). Besides, the level of tumor-infiltrating TIGIT^+^CD8^+^ T-cells have been remarkably associated inferior OS and relapse-free survival (RFS) of affected patients (HR = 2.17, 95% CI: 1.43–3.29, and *p*-value < 0.001, and HR = 1.89, 95% CI: 1.36–2.63, and *p*-value < 0.001, respectively). Also, there is a strong positive association between TIGIT expression with programmed cell death-1 (PD-1) expression in these patients (OR = 1.71, 95% CI: 1.10–2.68, and *p*-value = 0.02). In summary, increased TIGIT expression and increased infiltration of TIGIT^+^CD8^+^ T-cells can substantially worsen the prognosis of patients with solid cancers. Besides, concerning the observed strong association between TIGIT and PD-1, ongoing clinical trials, and promising preclinical results, PD-1/TIGIT dual blockade can potentially help overcome the immune-resistance state seen following monotherapy with a single immune checkpoint inhibitor in patients with solid cancers.

## 1. Introduction

Solid cancers have remained one of the daunting public health burdens worldwide [1]. Recent advances in immunotherapy have paved the way for introducing novel treatments for patients with solid tumors. However, the undesirable response rates of immunotherapeutic approaches have been a major obstacle for their translation into clinical practice for patients with solid cancers [2]. Thus, a better understanding of the cross-talk between immune cells and tumoral cells might provide valuable insights to ameliorate the response rates of affected patients to immunotherapeutic approaches.

The immunosuppressive tumor microenvironments of solid cancers might be critical in inhibiting the stimulation of effector immune cells [3,4]. Indeed, the immunosuppressive tumor microenvironment can exhaust effector immune cells and prevent tumor rejection [5]. Despite the ever-increasing FDA-approved immune checkpoint inhibitors for cancer patients, they have not completely restored the anti-tumoral immune responses in all solid cancers [6]. Therefore, there is a need to identify novel immune checkpoints in patients with solid cancers to restore the anti-tumoral immune responses.

The PD-1/programmed death-ligand 1 (PD-L1) inhibitory axis has been one of the well-studied inhibitory immune checkpoint axes in cancers; thus, targeting this axis via monoclonal antibodies was among the attempts to stimulate anti-tumoral immune responses [7]. This axis can be established between immune and tumor cells and can substantially contribute to immunosuppressive tumor microenvironment development [8]. Although monoclonal antibodies targeting this inhibitory axis have been promising for some solid cancers, like triple-negative breast cancer, they have not yielded meaningful results in other solid cancers, like glioblastoma [9,10]. The low response rate of some patients to anti-PD-1 might be stemmed from the fact that other inhibitory immune checkpoint molecules can also regulate anti-tumoral immune responses. Deng et al. have shown that CD8^+^ T-cell subpopulations widely express PD-1, cytotoxic T-lymphocyte-associated protein 4 (CTLA-4), T-cell immunoglobulin mucin-3 (TIM-3), and lymphocyte activation gene-3 (LAG-3), and TIGIT [11]. Kim et al. have indicated that the majority of exhausted CD8^+^ T-cells highly express CTLA-4, LAG-3, TIGIT, and TIM-3 [12]. Therefore, other inhibitory immune checkpoint molecules might contribute to maintaining the immunosuppressive tumor microenvironment following monotherapy with monoclonal antibodies targeting one inhibitory axis.

Firstly, Yu et al. have identified TIGIT as an inhibitory signal that can repress T-cell activation [13]. TIGIT can be overexpressed in CD8^+^ T-cells, regulatory T-cells (Tregs), CD4^+^ T-cells, and natural killer (NK) cells [6]. Zhang et al. have shown that the TIGIT blockade can enhance NK cell-mediated anti-tumoral immune responses and improves the response rates of monoclonal antibodies targeting the PD-1/PD-L1 axis [14]. Besides, Wu et al. have demonstrated that TIGIT can be overexpressed in tumor-infiltrating CD8^+^ and CD4^+^ T-cells, and there is a remarkable relationship between TIGIT with PD-1, LAG-3, and TIM-3 in the tumor-infiltrating CD4^+^ and CD8^+^ T-cells in animal models of head and neck squamous cell carcinomas [15]. Indeed, the co-expression of TIGIT with other inhibitory immune checkpoint inhibitors, e.g., PD-1 and V-domain immunoglobulin suppressor of T cell activation (VISTA), might provide ample opportunities to reverse the immune-resistance state implicated in the unfavorable response rate of immune therapy with one immune checkpoint inhibitor [16,17].

Herein, the current meta-analysis aims to systematically investigate the significance of TIGIT in patients with solid cancers. Besides offering an unbiased insight into the significance of TIGIT in patients with solid cancers, bridging the results of this study with the recent preclinical results that are discussed in the discussion section might provide ample opportunities to improve the response rate of solid cancer patients to immune checkpoint inhibitor-based therapies.

## 2. Material and Methods

This study was conducted according to the PRISMA statements [18].

### 2.1. The Strategy of the Systematic Search

The Web of Science, Embase, PubMed, and Scopus databases were systematically searched to obtain the peer-reviewed records published before 20 September 2020. For this purpose, the aforementioned databases were systematically searched with the following keywords: (“tumor” OR “tumour” OR “malignancy” OR “neoplasm” OR “neoplasia” OR “malignant” OR “carcinoma” OR “cancerous” OR “tumoral” OR “tumoural” OR “neoplastic”) and (“T-cell immunoglobulin and immunoreceptor tyrosine-based inhibitory motif (ITIM) domain” OR “T cell immunoglobulin and immunoreceptor tyrosine-based inhibitory motif (ITIM) domain” OR “T Cell Immunoreceptor With Ig and ITIM Domains” OR “T cell immunoglobulin and ITIM domain” OR “T-Cell Immunoreceptor With Ig and ITIM Domains” OR “T-cell immunoglobulin and ITIM domain” OR “V-Set and Transmembrane Domain-Containing Protein 3” OR “V-Set and Transmembrane Domain Containing 3” OR “V-Set and Immunoglobulin Domain-Containing Protein 9” OR “V-Set and Immunoglobulin Domain Containing 9” OR “VSIG9” OR “VSTM3” OR “WUCAM” OR “TIGIT”).

### 2.2. Study Selection and Data Extraction

Following the systematic search, the obtained records were reviewed in two phases. In phase I, the records were screened based on their titles/abstracts. In phase II, the full text of papers and their supplementary data were reviewed for consideration to be involved in the current study. Any disagreements were resolved via consulting with B.B and consensus.

### 2.3. Eligibility Criteria

Papers with the following eligibility criteria were included in the current study: (1) clinical studies, (2) studies with the objective of assessing TIGIT immune checkpoint in patients with solid cancers, (3) studies that evaluated the prognostic value of TIGIT immune checkpoint or the clinicopathological significance of TIGIT in patients with solid cancers, and (4) studies that were published in English.

### 2.4. Data Extraction

The following data were extracted from the included studies: (1) the first author, (2) the publication year, (3) the country, (4) the sample size, (5) male to female ratio, (6) median age, (7) high tumor stage/low tumor stage ratio, (8) the cancer therapy records of the patients, (9) TIGIT evaluation method, (10) TIGIT antibody ID, (11) the prognostic value of TIGIT immune checkpoint, (12) the clinicopathological significance of TIGIT, (13) the association between TIGIT and PD-1 immune checkpoints, and (14) the prognostic value of TIGIT^+^CD8^+^ tumor-infiltrating lymphocytes.

### 2.5. Assessing the Potential Risk of Bias among the Included Studies

To improve the transparency of the obtained results, we applied the Hayden et al. guideline to assess the quality of included prognostic studies [19]. We also applied the JBI critical appraisal checklist for evaluating the quality of studies concerning the clinicopathological significance of TIGIT [20].

### 2.6. Statistical Analysis

The analyses were performed by STATA16 (StataCorp, College Station, TX, USA). Random effect meta-analyses were conducted utilizing the REML [21]. The random-effect model was applied because there may be other unknown, unregistered/unpublished studies that we could not have access. The I-squared, H-squared, Tau-squared, and Cochran Q test statistics were performed to assess included studies’ heterogenicity. Regarding the I-squared, the value above 50% was considered as high heterogeneity, and the H-Squared = 1 was considered as homogeneity among included studies [22]. The funnel plots were provided to assess the asymmetry and publication bias. For the evaluation of bias, Egger’s and Begg’s tests were performed [23,24].

## 3. Results

### 3.1. Systematic Search

**Figure 1:**
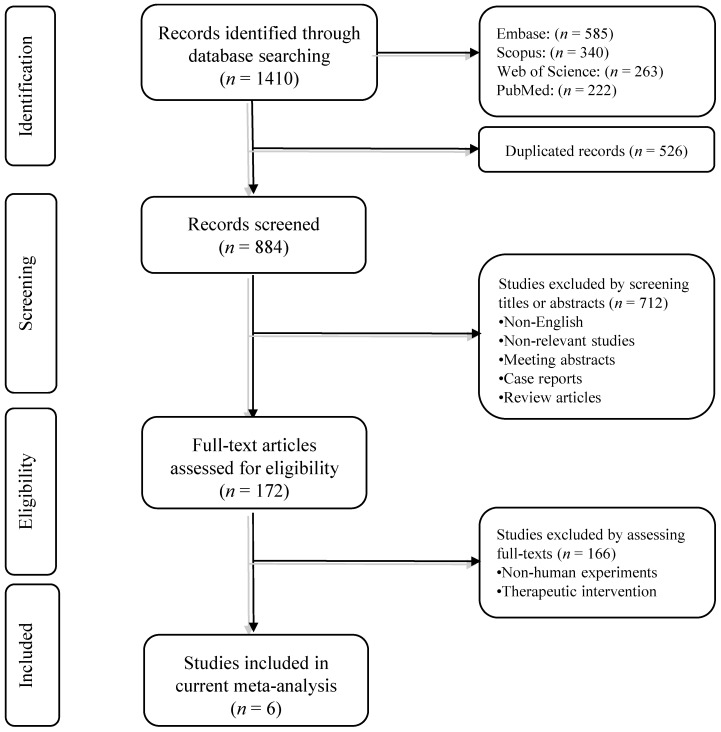
The flowchart of the study selection process.

Our systematic search retrieved 1410 records. After removing duplicated studies, 884 records remained. Based on screening the title/abstract of the records, 712 papers did not meet the aforementioned inclusion criteria. In phase II, the full text of the remaining 172 studies and their supplementary data were reviewed. Finally, six studies met the abovementioned criteria and were included in the quantitative synthesis. The flowchart of literature inclusion and exclusion is shown in Figure 1.

### 3.2. The Characteristic of Included Studies

The characteristic of the included studies is demonstrated in Table 1. The six clinical studies were published between 2018 and 2020. The studied solid cancers were esophageal squamous cell carcinoma [25], renal cell carcinoma [26], gastric adenocarcinoma [27], cutaneous melanoma [28], lung adenocarcinoma [29], and muscle-invasive bladder cancer [30]. Two studies only evaluated the clinicopathological significance of TIGIT in affected patients [26,27]. Except for the study by Lee et al., other included studied have ethical approvals [25,26,27,29,30]. The characteristics of the included studies are demonstrated in Table 1.

### 3.3. The Clinicopathological Significance of TIGIT

**Figure 2:**
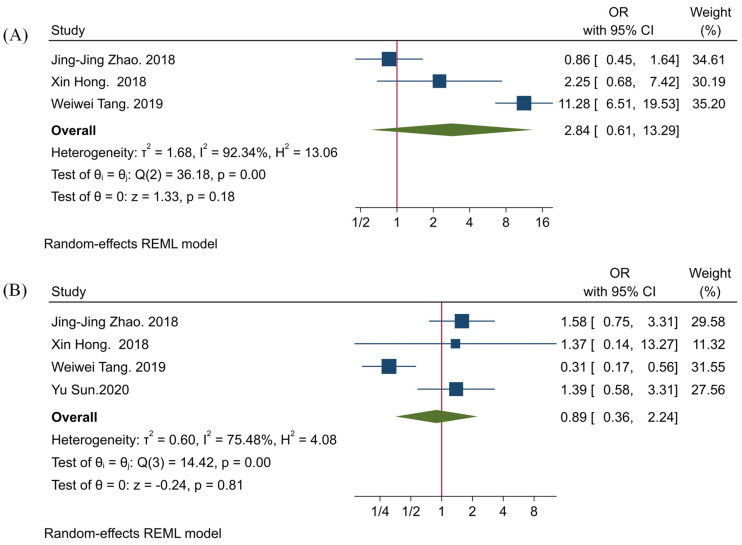
Forest plots for evaluating the association between TIGIT expression and clinicopathological characteristics of affected patients. (**A**) tumor size, and (**B**) tumor differentiation.

Our results have shown no statistically significant associations between TIGIT expression with tumor size and tumor differentiation (HR = 2.84, 95% CI: 0.61–13.29, *p*-value = 0.18, and HR = 0.89, 95% CI: 0.36–2.24, *p*-value = 0.81, respectively) (Figure 2).

### 3.4. The Association between TIGIT and PD-1

**Figure 3:**
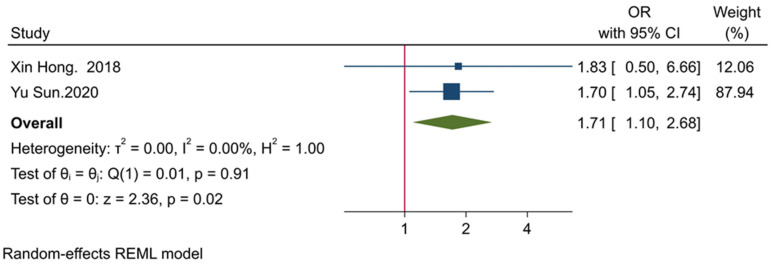
Forest plot for evaluating the association between the expression of TIGIT and PD-1.

Our study has demonstrated that there is a significant association between the expression of TIGIT and PD-1 (OR = 1.71, 95% CI: 1.10–2.68, and *p*-value = 0.02) (Figure 3). Besides, our results have indicated that there is no significant heterogeneity among the included studies (*p*-value = 0.91, I^2^ = 0.00%, and H^2^ = 1.00) (Figure 3).

### 3.5. The Prognostic Value of TIGIT

**Figure 4:**
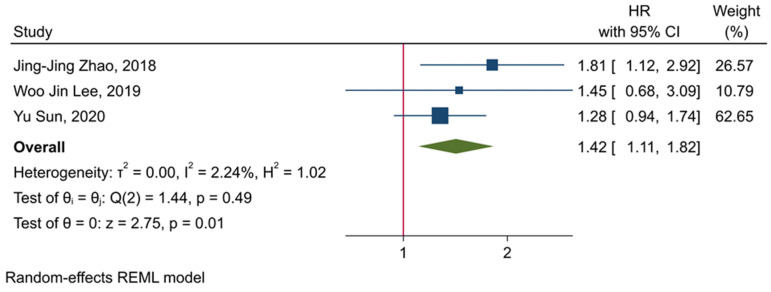
Forest plot for evaluating the prognostic value of TIGIT expression in determining the OS of affected patients.

Our results have shown that increased TIGIT expression is significantly associated with the inferior OS of affected patients (HR = 1.42, 95% CI: 1.11–1.82, and *p*-value = 0.01) (Figure 4). Also, our results have indicated that there is no significant heterogeneity among the included studies (*p*-value = 0.49, I^2^ = 2.24%, and H^2^ = 1.02) (Figure 4).

### 3.6. The Prognostic Value of Tumor-Infiltrating TIGIT^+^CD8^+^ T-Cells

**Figure 5:**
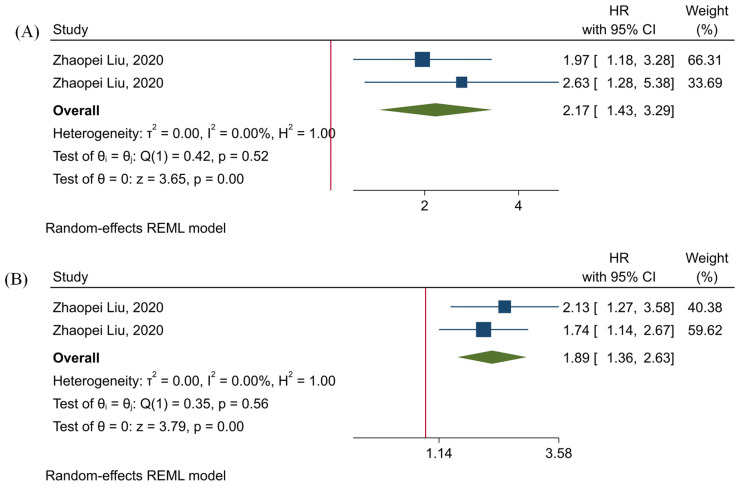
Forest plots for assessing the prognostic value of tumor-infiltrating TIGIT^+^CD8^+^ T-cells. (**A**) OS, and (**B**) RFS.

Our results have shown that the level of tumor-infiltrating TIGIT^+^CD8^+^ T-cells is significantly associated the inferior OS and RFS of affected patients (HR = 2.17, 95% CI: 1.43–3.29, and *p*-value < 0.001, and HR = 1.89, 95% CI: 1.3–2.63, and *p*-value < 0.001, respectively) (Figure 5). Besides, our results have indicated that there is no significant heterogeneity among the included studies (*p*-value = 0.52, I^2^ = 0.00%, and H^2^ = 1.00 in the case of OS, and *p*-value = 0.56, I^2^ = 0.00%, and H^2^ = 1.00 in the case of RFS) (Figure 5).

### 3.7. Assessing Potential Bias among the Included Studies

The summaries of the evaluated quality of the included studies are demonstrated in Table 2 and Table 3. The main risk area has been about addressing the cofounders, which is stemmed from the nature of non-randomized studies. However, the quality of the included studies in other areas has been acceptable (Table 2 and Table 3). Overall, the quality of the included studies is acceptable.

### 3.8. Evaluating Publication Bias

**Figure 6:**
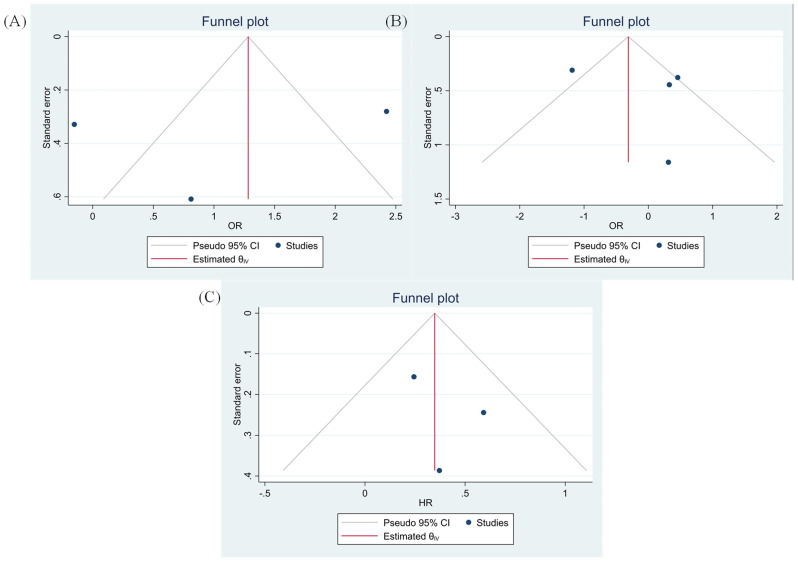
Funnel plots based on TIGIT expression (**A**) tumor size (Egger test *p*-value = 0.769 and Begg’s test *p*-value = 0.149), (**B)** tumor differentiation (Egger test *p*-value = 0.579 and Begg’s test *p*-value = 0.367) and (**C**) OS (Egger test *p*-value = 0.500 and Begg’s test *p*-value = 0.500).

The Egger’s and Begg’s tests have demonstrated no significant publication bias (Figure 6).

## 4. Discussion

Although multiple studies have investigated the prognostic value and clinicopathological significance of TIGIT in patients with solid cancers, their results remain inconclusive [25,26,27,28,29,30]. The current study is the first meta-analysis to study the significance of TIGIT in patients with solid cancers.

As a novel inhibitory immune checkpoint, TIGIT competes with co-stimulatory CD226 to bind with CD155 and CD112. TIGIT has a central role in attenuating immune responses. TIGIT can substantially impair dendritic cells via upregulating IL-10 expression [13]. IL-10 has been associated with decreased function of dendritic cells in developing anti-tumoral immune responses [31]. Besides, TIGIT can substantially inhibit NK cell-mediated anti-tumoral immune responses. Stanietsky et al. have shown that TIGIT can inhibit NK cells cytotoxicity, and its blockade can upregulate interferon-γ (IFN-γ) [32]. Meng et al. have reported that TIGIT^+^NK cells express fewer IFN-γ and tumor necrosis factor-α (TNF-α) than TIGIT-NK cells, and the high expression of TIGIT is associated with decreased function of NK cells and immune evasion of tumoral cells [33]. Moreover, TIGIT has been implicated in impairing the anti-tumoral immune responses of CD8^+^ T-cells. Weiling et al. have shown that TIGIT^+^ CD8 T-cells are functionally exhausted, and their proliferation is limited. Besides, tumoral cells can upregulate CD155 and facilitate CD8^+^ T-cells inactivation via the TIGIT/CD155 inhibitory axis. Also, silencing tumoral CD155 has been associated with increased expression of IFN-γ and restored metabolism of T-cells [34]. Furthermore, Zhou et al. have shown that tumor-intrinsic TIGIT can substantially inhibit NK-cells and CD8^+^ T-cells-mediated anti-tumoral immune responses and pave the way for tumor growth in vivo [35]. Consistent with these preclinical findings, our results have demonstrated that high expression of TIGIT is significantly associated with inferior OS in patients with solid cancers (HR = 1.42, 95% CI: 1.11–1.82, and *p*-value = 0.01). Besides, our results have indicated that increased infiltration of tumor-infiltrating TIGIT^+^CD8^+^ T-cells is remarkably associated inferior OS and RFS (HR = 2.17, 95% CI: 1.43–3.29, and *p*-value < 0.001, and HR = 1.89, 95% CI: 1.36–2.63, and *p*-value < 0.001, respectively). Therefore, TIGIT is a pivotal inhibitory immune checkpoint that its high expression in tumoral cells and cells residing in the tumor microenvironment can remarkably attenuate anti-tumoral immune responses.

Our results have indicated that the high expression of TIGIT might not be statistically associated with poor tumor differentiation and increased tumor size (both *p*-values > 0.05). However, the current meta-analysis has shown a significant association between TIGIT and PD-1 in patients with solid cancers (OR = 1.71, 95% CI: 1.10–2.68, and *p*-value = 0.02). Wu et al. have reported that anti-TIGIT monoclonal antibodies can substantially decrease arginase-1 transcription levels in myeloid-derived suppressor cells (MDSCs) [15]. Liu et al. have reported that anti-PD-1 monoclonal antibodies and PD-1 silencing of MDSCs can also substantially decrease arginase-1 expression in MDSCs [36]. Arginase-1 is an inhibitory factor that depletes L-arginine from the tumor microenvironment and induces T cell anergy [37]. Dufait et al. have shown that arginase-1 expression is elevated in tumor and in vitro generated MDSCs, and its inhibition can substantially increase the proliferation of T cells and decrease tumor volume in animal models [38]. MDSCs can remarkably express arginase-1 and contribute to the development of the immunosuppressive tumor microenvironment. Heuvers et al. have shown a remarkable correlation between arginase-1 and peripheral blood MDSCs [39]. Consistent with this, Ren et al. have indicated that early-stage MDSCs of peripheral blood and tumor-infiltrating early-stage MDSCs can upregulate arginase-1 [40]. Besides, the co-culture of MDSCs with tumoral cells can upregulate arginase-1 expression in MDSCs [41]. Although the exact mechanism of TIGIT and PD-1 co-expression has not been exactly identified in tumors, the currently available evidence indicates that TIGIT and PD-1 can both increase arginase-1 activity, which can ultimately lead to T cells anergy and the development of immunosuppressive tumor microenvironment. However, further studies are needed to elucidate the underlying mechanisms for the co-expression of TIGIT and PD-1 in cancer.

In line with our obtained results, Hung et al. have shown that TIGIT and PD-1 co-expression are substantially upregulated in tumor-infiltrating CD8^+^ T-cells in glioma animal models [16]. In patients with bladder cancer, TIGIT expression has also been predominantly co-expressed on PD-1^+^ tumor-infiltrating CD8^+^ T-cells. Moreover, TIGIT blockade can improve the efficacy of anti-PD-1 therapy and promote the stimulation of tumor-infiltrating CD8^+^ T-cells in patients with bladder cancer [42]. Besides, TIGIT/PD-1 dual blockade has remarkably improved anti-tumoral immune responses and promoted tumor rejection in lymphoma animal models [10]. Furthermore, the dual blockade of the immune checkpoint axes of PD-1 and TIGIT has been superior in rejecting melanoma and non-small-cell lung carcinoma [43,44]. Although our results have only shown a strong positive association between PD-1 and TIGIT expression, the results of the aforementioned studies indicate that dual PD-1/TIGIT blockade is superior in tumor rejection, which might imply the strong positive association between PD-1 and TIGIT in the tumor microenvironment of solid cancers. Also, multiple ongoing clinical trials investigate the efficacy of dual TIGIT/PD-1 blockade in various solid cancers (Table 4).

The current study has some strengths. First, despite the various types of studied solid cancers in the included studies, our presented results have been homogenous, and there has been no significant heterogeneity that can jeopardize the obtained results regarding TIGIT significance. Second, consistent with preclinical findings, the current meta-analysis has highlighted the remarkable association between TIGIT and PD-1 on the clinical scale. Third, concerning the fact that TIGIT can be overexpressed in various immune cells, the current study has demonstrated the prognostic value of tumor-infiltrating TIGIT^+^CD8^+^ T-cells in patients with solid cancers. Fourth, our study has bridged the preclinical findings with the clinical findings. However, this study has some limitations, as well. Only papers published in English have been included in the current study. The second limitation of our study stems from the very nature of non-randomized clinical trial studies, in which all the confounder variables might not be addressed.

## 5. Conclusions

The current study has shown that the elevated expression of TIGIT in the tumor microenvironment is associated with inferior OS. Besides, the increased infiltration of tumor-infiltrating TIGIT^+^CD8^+^ is associated with the worsened OS and RFS of affected patients. Concerning the observed results regarding the strong positive association between TIGIT and PD-1 and the promising results of preclinical studies, dual PD-1/TIGIT blockade can substantially help overcome the immune-resistance state in solid cancers. Consistent with the current evidence and the current trend in the clinical trials for solid cancer patients, dual PD-1/TIGIT blockade can potentially ameliorate the response rate of solid cancer patients to immune checkpoint inhibitors.

## Figures and Tables

**Table 1:** The characteristics of included studies.

No.	The First Author and Publication Year	Country	Sample Size	Male/Female Ratio	Median Age	High Stage/Low Stage	Cancer Type	Endpoint	Cancer Therapy Record	TIGIT Evaluation Method	TIGIT Antibody ID
1	Zhao, 2018	China	154	4.13	55	0.57	Esophageal squamous cell carcinoma	OS	No chemotherapy/immunotherapy before surgery	IHC	MBSA43
2	Hong, 2018	China	60	1.72	55.6	0.07	Renal cell carcinoma	Clinicopathological association	No radiotherapy, chemotherapy, and biological therapy before surgery	IHC	N/a
3	Tang, 2019	China	441	1.25	N/a	0.36	Gastric adenocarcinoma	Clinicopathological association	No neoadjuvant chemotherapy/radiotherapy before surgery	IHC	ab233404
4	Lee, 2019	South Korea	124	1.21	N/a	2.12	Cutaneous melanoma	OS	N/a	IHC	TG1
5	Sun, 2020	China	334	1.19	56	0.39	Lung adenocarcinoma	OS	N/a	IHC	MBS20013451
6	Liu, 2020	China	141	4.87	62	0.62	Muscle-invasive bladder cancer	OS/RFS	One hundred nineteen patients of these cohorts received adjuvant cisplatin-based chemotherapy.	IHC	ab243903
7	Liu, 2020	China	118	6.37	62	1.68	Muscle-invasive bladder cancer	OS/RFS		IHC	ab243903

Abbreviations: OS: Overall survival, RFS: Relapse-free survival, IHC: Immunohistochemistry, and N/a: Not available.

**Table 2:** Evaluating the potential bias in the included prognostic studies based on the Hayden et al. statements.

No.	The First Author and Publication Year	Study Participation	Study Attrition	Prognostic Factor Measurement	Outcome Measurement	Confounding Measurement and Account	Analysis
1	Zhao, 2018	***	***	***	***	**	***
2	Lee, 2019	**	***	***	***	*	***
3	Sun, 2020	***	***	***	***	**	***
4	Liu, 2020	***	***	***	***	**	***

*: bias might be present; **: bias might be partly present; ***: bias might not be present.

**Table 3:** Evaluating the potential bias in the included studies based on the JBI critical appraisal checklist.

Major Components	Hong, 2018	Tang, 2019
1. Were the criteria for inclusion in the sample clearly defined?	Yes	Yes
2. Were the study subjects and the setting described in detail?	Yes	Yes
3. Was the exposure measured in a valid and reliable way?	Yes	Yes
4. Were objective, standard criteria used for measurement of the condition?	Yes	Yes
5. Were confounding factors identified?	Unclear	Unclear
6. Were strategies to deal with confounding factors stated?	Unclear	Unclear
7. Were the outcomes measured in a valid and reliable way?	Yes	Yes
8. Was appropriate statistical analysis used?	Yes	Yes

**Table 4:** The current clinical trials for targeting TIGIT immune checkpoint.

No.	Intervention	Mechanism of Action	Cancer Type	Clinical Trial Phase	Study Start Date	The Status	Country	Clinicaltrials.gov Identifier
1	ASP8374, ASP8374 + Pembrolizumab	an anti-TIGIT mAb + an anti-PD-1 mAb	Advanced solid tumors	Phase 1	8-September-17	Active, not recruiting	International	NCT03260322
2	Tiragolumab, Tiragolumab + Atezolizumab	an anti-TIGIT mAb + an anti-PD-L1 mAb	Advanced/Metastatic tumors	Phase 1	23-May-16	Recruiting	International	NCT02794571
3	BGB-A1217+ Tislelizumab	an anti-TIGIT mAb	Advanced solid tumors	Phase 1	26-August-19	Recruiting	International	NCT04047862
4	Tiragolumab + Atezolizumab	an anti-TIGIT mAb + an anti-PD-L1 mAb	NSCLC	Phase 2	10-August-18	Active, not recruiting	International	NCT03563716
5	AB154 + Zimberelimab	an anti-TIGIT mAb + an anti-PD-1 mAb	Advanced solid tumors	Phase 1	12-September-18	Recruiting	International	NCT03628677
6	vibostolimab, vibostolimab + Pembrolizumab	an anti-TIGIT mAb + an anti-PD-1 mAb	Advanced solid tumors	Phase 1	13-December-16	Recruiting	International	NCT02964013
7	BMS-986207, BMS-986207 + Nivolumab	an anti-TIGIT mAb + an anti-PD-1 mAb	Broad solid tumors	Phase 1/2	29-November-16	Active, not recruiting	International	NCT02913313
8	Atezolizumab, Atezolizumab + Tiragolumab	an anti-PD-L1 mAb + an anti-TIGIT mAb	SCLC	Phase 3	4-February-20	Recruiting	International	NCT04256421
9	Tiragolumab + Atezolizumab	an anti-TIGIT mAb + an anti-PD-L1 mAb	NSCLC	Phase 3	4-March-20	Recruiting	International	NCT04294810
10	Tiragolumab+ Atezolizumab	an anti-TIGIT mAb + an anti-PD-L1 mAb	Gastric cancer	Phase 1/2	13-October-17	Recruiting	International	NCT03281369
11	AB154+ zimberelimab	an anti-TIGIT mAb + an anti-PD-1 mAb	NSCLC	Phase 2	10-February-20	Recruiting	International	NCT04262856
13	BMS-986207 + Nivolumab + COM701	an anti-TIGIT mAb + an anti-PD-1 mAb + an anti-PVRIG mAb	Advanced solid tumors	Phase 1/2	31-August-20	Recruiting	United States	NCT04570839
14	Atezolizumab, Atezolizumab + Tiragolumab	an anti-PD-L1 mAb + an anti-TIGIT mAb	Esophageal squamous cell carcinoma	Phase 3	28-September-20	Recruiting	International	NCT04543617
15	Tislelizumab, Tislelizumab + BGB-A1217	an anti-TIGIT mAb+ an anti-PD-1 mAb	Cervical cancer	Phase 2	25-January-21	Not yet recruiting	China	NCT04693234
17	M6223	an anti-TIGIT mAb	Metastatic solid tumors	Phase 1	10-July-20	Recruiting	International	NCT04457778
18	IBI939	an anti-TIGIT mAb	Advanced NSCLC	Phase 1	6-June-21	Not yet recruiting	China	NCT04672369
19	IBI939 + Sintilimab	an anti-TIGIT mAb + an anti-PD-1 mAb	Advanced lung cancer	Phase 1	28-January-21	Not yet recruiting	China	NCT04672356

Abbreviations: TIGIT: T cell immunoreceptor with Ig and ITIM domains, PD-1: Programmed cell death protein 1, PD-L1: Programmed death-ligand 1, NSCLC: Non-small cell lung cancer, SCLC: Small-cell lung cancer, and mAb: Monoclonal antibody.

## Data Availability

Not applicable.
